# Electrochemical Endpoint Determination and Machine-Learning Prediction of Pickling Time for Hot-Rolled Automotive High-Strength Steel

**DOI:** 10.3390/ma19183974

**Published:** 2026-09-18

**Authors:** Zhou Xu, Jianfei Xu, Dongdong Ye, Changdong Yin, Yiwen Wu, Qiang Liu, Xinchun Huang, Longhai Liu, Jianjun Chen

**Affiliations:** 1 School of Electrical and Automation, Wuhu Vocational Technical University, Wuhu 241006, China; 101043@whit.edu.cn (Z.X.);; 2 Opening Project of Anhui Key Laboratory of Electric Drive and Control, School of Electrical Engineering, Anhui Polytechnic University, Wuhu 241000, China; 3 School of Intelligent Manufacturing, Wuhu University, Wuhu 241008, China; 4 School of Artificial Intelligence, Anhui Polytechnic University, Wuhu 241000, China; 5 Aviation Industry Corporation Huadong Photoelectric Co., Ltd., Wuhu 241003, China; 6 School of Mechanical and Power Engineering, East China University of Science and Technology, Shanghai 200237, China; 7 Institute of Intelligent Manufacturing, Wuhu Vocational Technical University, Wuhu 241006, China; 8 Anhui Yingrui Excellent Material Technology Co., Ltd., Hengshan Avenue, Wuhu 241000, China; 9 Key Laboratory of Opto-Electronics Information Technology, Ministry of Education, School of Precision Instruments and Opto-Electronics Engineering, Tianjin University, Tianjin 300072, China

**Keywords:** hot-rolled high-strength steel, oxide scale, acid pickling, electrochemical endpoint detection, potential derivative, PSO-ELM, machine learning

## Abstract

**Highlights:**

- A potential-derivative near-zero method was developed to identify the pickling endpoint.
- A derivative threshold of −5 × 10^−4^ V/s was established as an operational criterion for endpoint detection.
- PSO-ELM achieved the best repeated grouped cross-validation performance, with R^2^ = 0.85 ± 0.05 and RMSE = 9.65 ± 1.48 s.

**Abstract:**

Accurate determination and prediction of pickling time are essential for preventing under-pickling and over-pickling and for improving the surface quality of hot-rolled high-strength steel. In this study, an electrochemical endpoint detection method combined with hybrid machine-learning models was developed to predict the pickling time of hot-rolled automotive high-strength steel. The variation in open-circuit potential during hydrochloric-acid pickling was monitored using an electrochemical workstation, and a potential-derivative near-zero method was proposed to determine the completion of oxide-scale removal. Based on repeated experiments under ten representative process conditions, a potential-derivative threshold of −5 × 10^−4^ V/s was adopted as the operational criterion for identifying the pickling endpoint. The effects of oxide-scale thickness, HCl concentration, pickling temperature, and accelerator concentration on pickling time were subsequently investigated. To describe the nonlinear relationship between these variables and pickling time, BP, GA-BP, ELM, and PSO-ELM regression models were established. The 48-observation dataset was evaluated using leakage-free grouped nested six-fold cross-validation repeated ten times, with all observations from the same strip group kept within the same fold. Among the investigated models, PSO-ELM exhibited the best prediction performance, achieving R^2^ = 0.85 ± 0.05, MAE = 7.27 ± 1.01 s, MAPE = 0.14 ± 0.02, and RMSE = 9.65 ± 1.48 s. These quantities are regression-performance statistics and are not interpreted as the percentage prediction accuracy. The proposed endpoint criterion and regression framework provide a laboratory-scale basis for data-driven pickling-time estimation within the investigated material and process ranges, and broader industrial application requires validation using larger multi-grade production datasets.

## 1. Introduction

Automotive lightweighting is an important route to reduce energy consumption and emissions while maintaining safety and recyclability [1,2]. High-strength steels are widely used because they provide favorable mechanical performance at a reduced component mass [3,4]. The quality of hot-rolled strip directly affects subsequent cold rolling and forming performance. Dimensional and shape-control technologies have substantially improved width, thickness, and flatness accuracy [5,6], while advances in steelmaking, continuous casting, and hot rolling have improved mechanical properties [7]. Surface quality therefore remains a critical issue. Because automotive high-strength steel is rolled and coiled at elevated temperatures, oxide scale with spatially varying thickness and composition forms on the strip surface and must be removed before cold rolling [8,9,10].

After final rolling, the hot-rolled strip is rapidly cooled from the elevated temperature. According to the Fe–O phase diagram [11], the scale may contain FeO adjacent to the substrate, Fe_3_O_4_ in the intermediate region, and Fe_2_O_3_ near the outer surface. Scale thickness can vary from several to hundreds of micrometers depending on processing history [12,13,14,15,16,17]. Mechanical, chemical, and combined mechano-chemical methods are available for oxide-scale removal [18,19,20,21,22], and hydrochloric-acid pickling remains widely used for hot-rolled strips. Inadequate pickling leaves residual scale, whereas excessive pickling can attack the steel substrate and generate surface defects. Accurate endpoint control is therefore important for both surface quality and process stability.

Precise control of pickling requires quantitative consideration of both oxide-scale condition and process parameters. A key input is oxide-scale thickness, but conventional destructive measurements provide only local information. In our previous study, terahertz nondestructive testing (NDT) combined with hybrid machine learning approaches was developed for nondestructive evaluation of thin oxide-scale thickness [23]. That work focused on oxide-scale thickness characterization rather than pickling-time prediction. In the present study, oxide-scale thickness is treated as one input together with acid concentration, pickling temperature, and accelerator concentration, while the electrochemically determined pickling time is used as the regression target.

Machine-learning methods have increasingly been used to describe nonlinear relationships in steel-processing systems. Data-driven models have been applied to predict width deviation and mechanical properties in hot-rolling processes [6,7], and previous work has also considered prediction of under-pickling defects [18]. Optimization-assisted neural models, including PSO-ELM approaches, have shown advantages in other nonlinear engineering-prediction problems [24,25]. However, these studies generally focus on product properties, geometric deviations, defect prediction, or other process outputs rather than on an electrochemically defined pickling endpoint. Accordingly, the main contribution of the present study is the integration of an electrochemical endpoint criterion with nonlinear regression models for pickling-time estimation, together with a comparison of BP, GA-BP, ELM, and PSO-ELM under the same dataset and evaluation framework. In contrast to Ref. [23], which treated oxide-scale thickness itself as the prediction target, the present framework uses measured scale thickness as a material-state input and couples it with three controllable pickling variables to predict an electrochemically defined process endpoint. This distinction is central to the novelty of the present work because the endpoint detection method and the regression model form a single process-control chain rather than two independent characterization tasks.

## 2. Materials and Methods

### 2.1. Experimental Procedures

**Figure 1:**
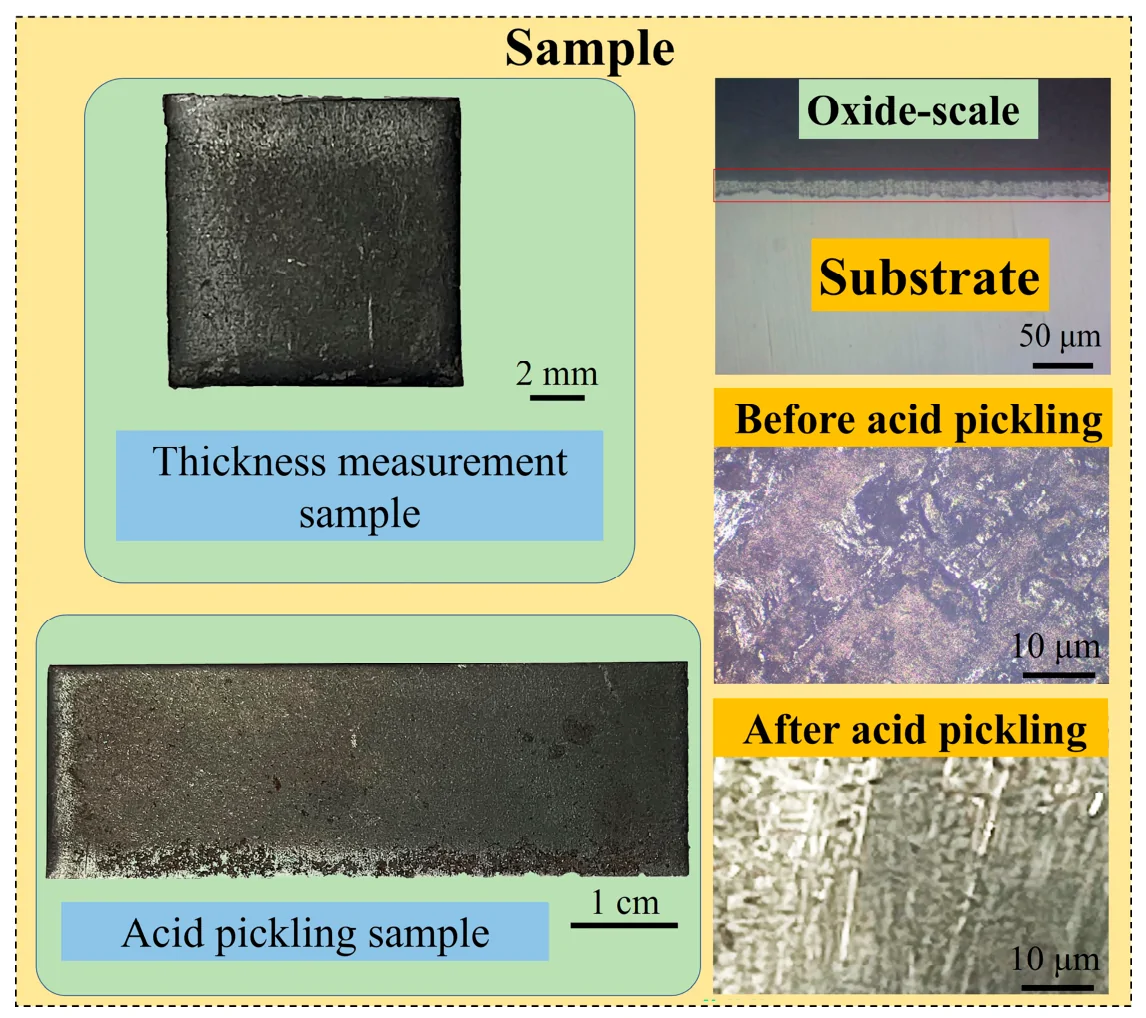
Specimen geometry and sampling locations for oxide-scale thickness measurement.

The investigated material was a commercial hot-rolled DP600 automotive dual-phase steel produced by China Baowu Iron & Steel Group Co., Ltd. (Shanghai, China) The nominal chemical composition (wt.%) was 0.08 C, 0.28 Si, 1.55 Mn, 0.014 P, 0.006 S, 0.18 Cr, and 0.035 Al, with Fe as the balance. The strip was finish rolled at 870 ± 10 °C and coiled at 610 ± 15 °C. Thickness measurement and pickling specimens were taken from the edge and middle regions at the head and tail positions of the strip, as shown in Figure 1. The thickness-measurement specimens were 10 mm × 10 mm × 3 mm, and the pickling specimens were 60 mm × 20 mm × 3 mm. Cross-sectional specimens were mounted, ground, and polished before examination. A 4XC-PC optical microscope (Shangguang Fifth Factory, Shanghai, China; ×200) was used for preliminary observation of oxide-scale morphology. Quantitative thickness measurements used for machine-learning inputs were obtained using a Hitachi SU3500 scanning electron microscope (Hitachi, Tokyo, Japan) operated at 15 kV and a working distance of approximately 10 mm. For each strip location, three independently prepared cross-sections were examined and ten approximately equally spaced thickness measurements were obtained from each cross-section. The reported range represents the minimum-to-maximum span of the measurements, and the largest measured value was used as the conservative scale-thickness input because complete pickling is governed by the locally thickest scale region. Repeated cross-sectional measurements gave a typical thickness standard deviation of approximately 0.8–1.0 μm. Before pickling, specimens were ultrasonically cleaned in anhydrous ethanol and dried. The cut edges were sealed with E7 adhesive, leaving the defined working-electrode surface exposed so that edge dissolution did not dominate the measured open-circuit potential.

**Figure 2:**
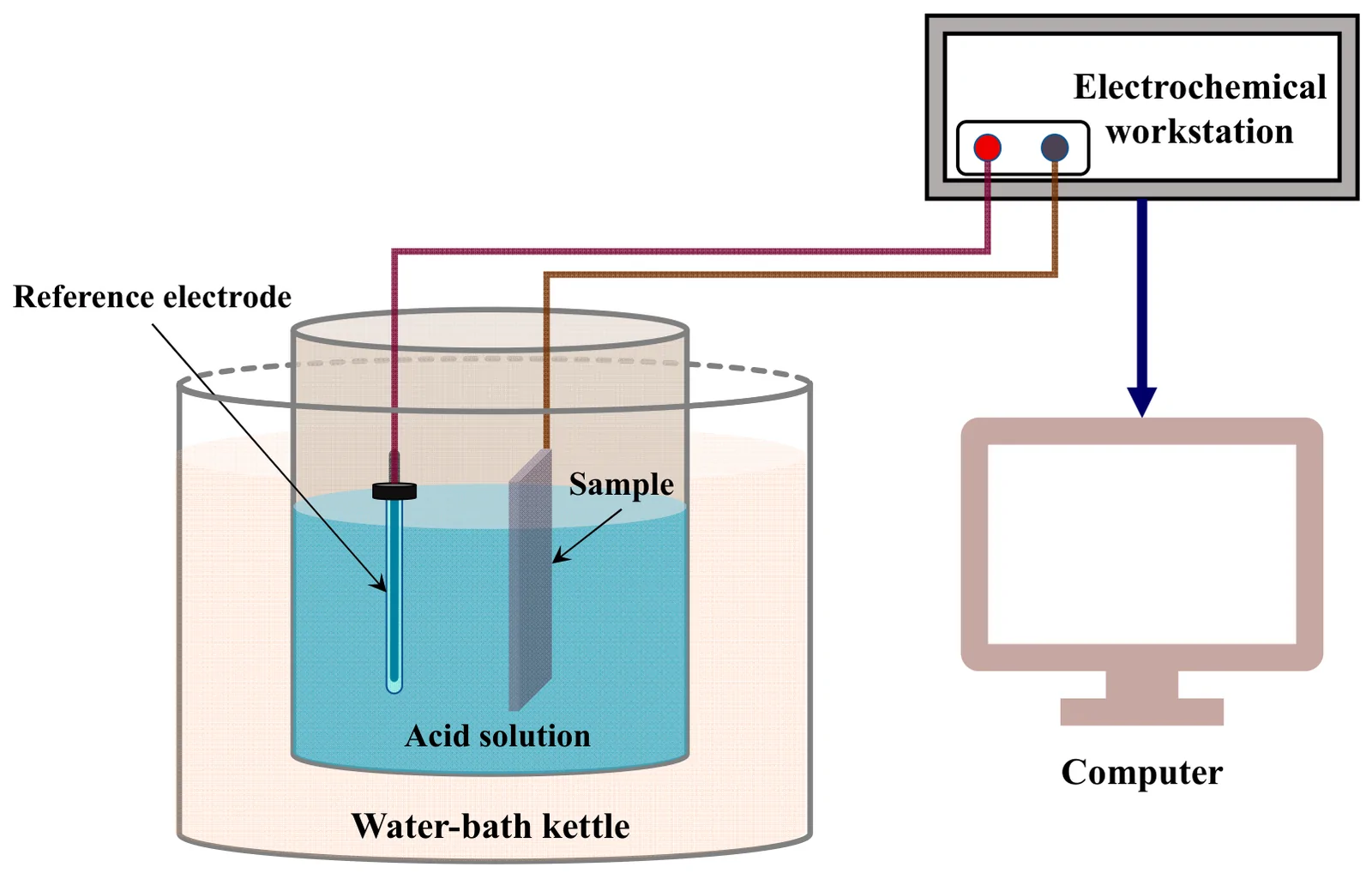
Schematic of the electrochemical pickling setup.

Open-circuit potential during pickling was recorded using a CHI604B electrochemical workstation. The measurement used a two-electrode configuration, with the steel specimen as the working electrode and an Ag/AgCl electrode as the reference electrode (Figure 2). The pickling solution was prepared by diluting analytical-grade hydrochloric acid (36–38 wt.% stock solution) with deionized water. HCl concentration in this study is reported as a mass fraction (wt.%). A commercial hydrochloric-acid pickling accelerator (PA51-A, Wuhan Research Institute of Materials Protection Co., Ltd., Wuhan, China), whose principal active component is sodium dodecylbenzene sulfonate, was added at 0, 1, or 3‰ by mass of the final solution. Each test used 500 mL of freshly prepared solution without forced mechanical agitation, and the specimen was immersed vertically. The tested HCl concentrations were 6, 10, and 14 wt.%, and the temperatures were 75, 80, and 85 °C (Table 1). Solution temperature was maintained within ±0.5 °C using a thermostatic water bath.

### 2.2. Potential Derivative Near-Zero Method

Pickling time was used as the response variable for evaluating the pickling process. Insufficient pickling leaves residual oxide scale, whereas excessive pickling can attack the steel substrate and adversely affect surface quality. Common endpoint methods include visual observation, mass-loss measurement, and electrochemical-potential monitoring. Visual inspection is operator-dependent, and mass-loss measurements are inconvenient for rapid endpoint determination. By contrast, the electrochemical potential changes as the multilayer oxide scale dissolves and approaches a comparatively stable level after the substrate becomes exposed. The potential signal therefore provides a practical basis for identifying the completion of oxide-scale removal.

To identify the endpoint more directly, the derivative of the pickling-potential curve was analyzed. As the potential approaches a stable level, its derivative should theoretically approach zero. In practice, signal fluctuations caused by the heterogeneous scale and the pickling environment mean that the derivative does not remain exactly zero immediately after scale removal. Waiting for an exact zero derivative can therefore prolong exposure and promote over-pickling. For this reason, a potential-derivative near-zero criterion was adopted, and the threshold was determined by relating the derivative value to microscopic observations of the specimen surface at different pickling times.

Open-circuit potential was recorded at 1 s intervals. To suppress high-frequency instrumental noise while preserving the transition associated with oxide-scale removal, the discrete potential series was smoothed using a second-order Savitzky–Golay filter with a five-point window. The potential derivative was then calculated by the central finite-difference expression ${\left(\frac{dE}{dt}\right)}_{i}=\;\frac{E_{i+1}\;-\;E_{i-1}}{2\Delta t}$, with Δ*t* = 1 s, and is reported in V/s. Where $E_{i}$ represents the *E* value at the *i*-th moment, ${\left(\frac{dE}{dt}\right)}_{i}$ is the rate of change at time $t_{i}$, and Δt is the time interval between two adjacent time points.

The operational endpoint was defined as the first time point at which $\frac{dE}{dt}$ reached or exceeded the prescribed threshold and remained at or above it for five consecutive samples. Requiring five consecutive samples prevents a single noise excursion or transient caused by scale detachment from being interpreted as the endpoint. The pickling-time label supplied to the machine-learning models was the elapsed time satisfying this complete criterion.

The threshold was validated under ten representative combinations spanning the investigated ranges of HCl concentration, temperature, and accelerator concentration. Each condition was tested in triplicate, and microscopy-based completion time and the corresponding derivative value were summarized as mean ± standard deviation. For the representative time-series experiment, residual oxide scale was quantified by background-normalized grayscale segmentation of the micrographs after excluding annotations and scale bars. A residual-scale area fraction ≤ 3% was used as the microscopy completion criterion.

### 2.3. Modeling Methods

#### 2.3.1. GA-BP Model

**Figure 3:**
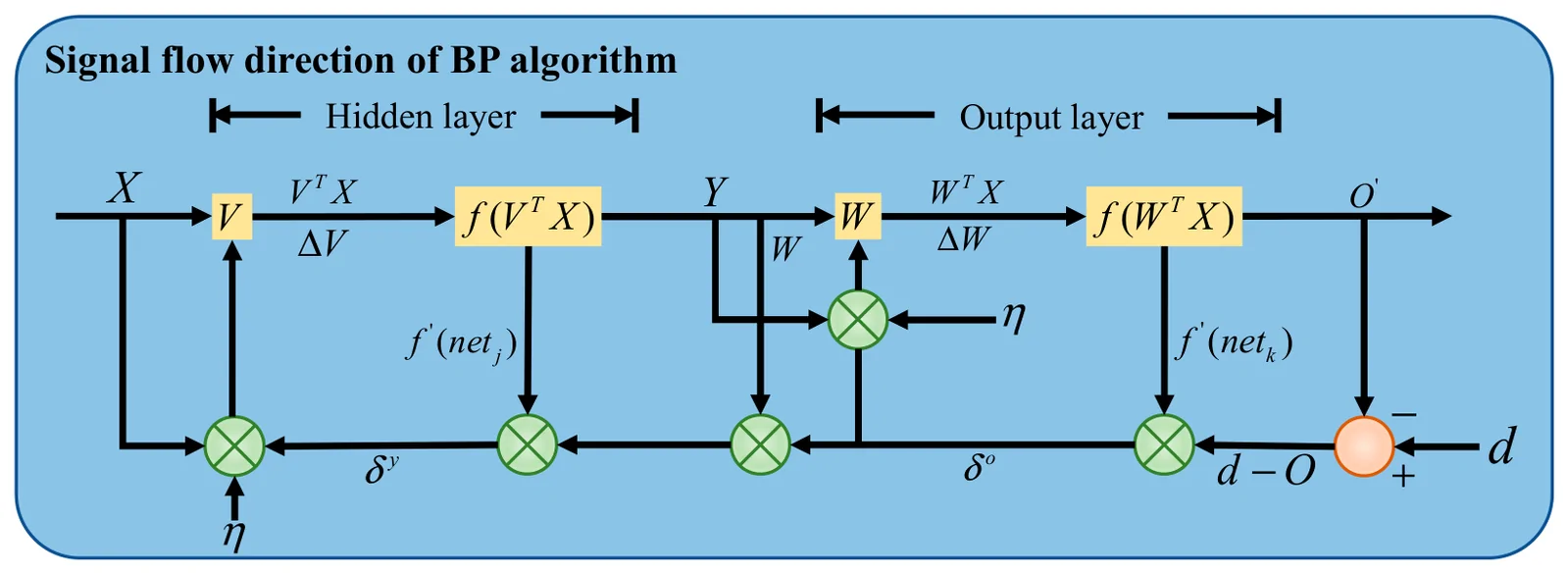
Schematic of signal propagation in the BP neural network.

The back-propagation (BP) neural network consists of a forward-propagation stage and a back-propagation stage for error correction [26]. During training, the input variables are nonlinearly transformed through the hidden layer and propagated to the output layer. The prediction error is then back-propagated to update the network weights and biases until the stopping criterion is reached. The BP architecture used in this study is shown in Figure 3. BP networks are flexible and capable of approximating nonlinear relationships; however, their convergence can be slow, they may become trapped in local minima, and their performance can be sensitive to network architecture and random initialization.

**Figure 4:**
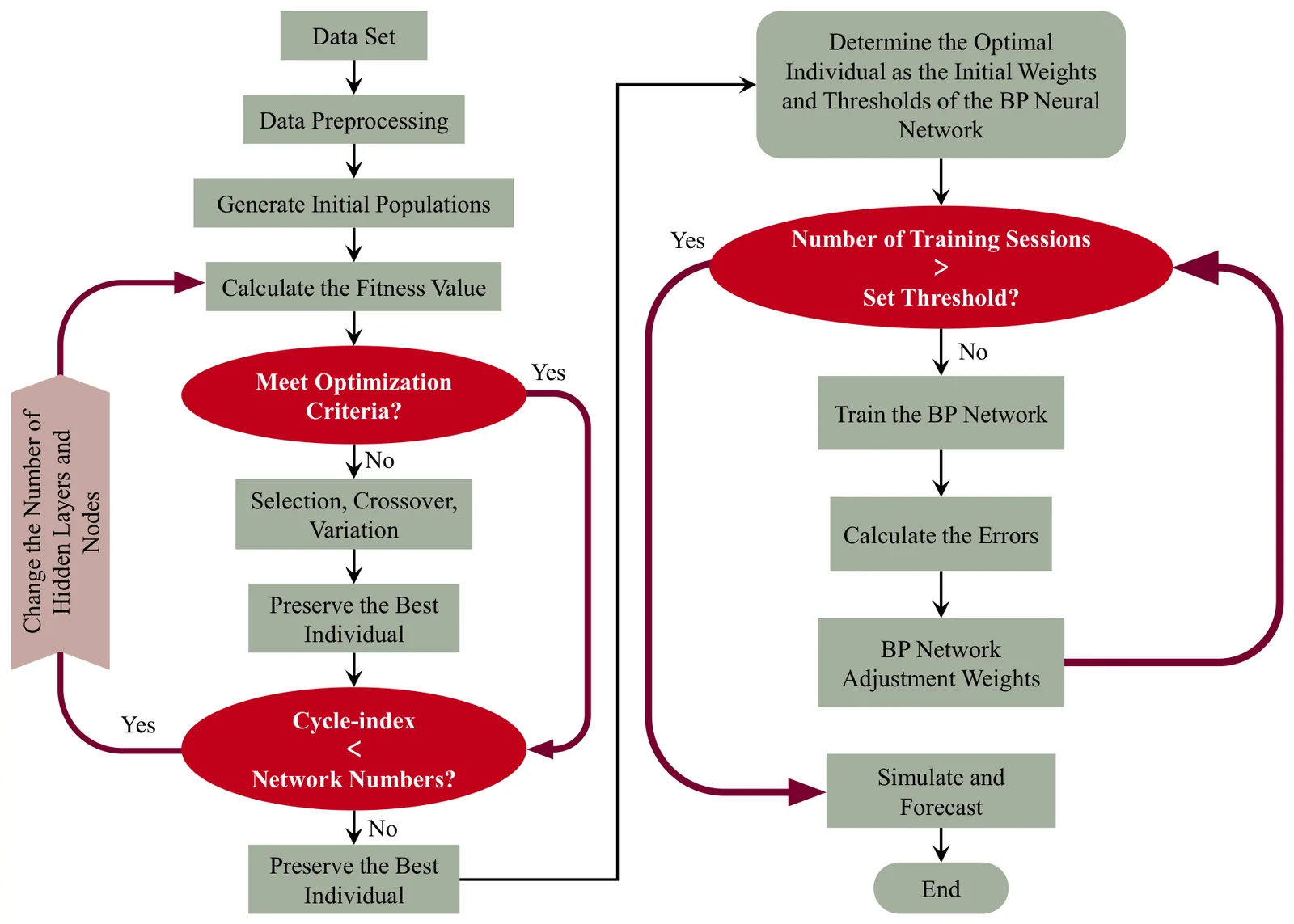
Flowchart of the GA-BP modeling procedure.

To improve robustness to the initial weights and biases, a genetic algorithm (GA) was used to optimize these parameters before BP training [27,28]. The GA searches the parameter space through iterative selection, crossover, and mutation according to a fitness criterion. The resulting GA-BP modeling workflow is shown in Figure 4.

#### 2.3.2. PSO-ELM Model

**Figure 5:**
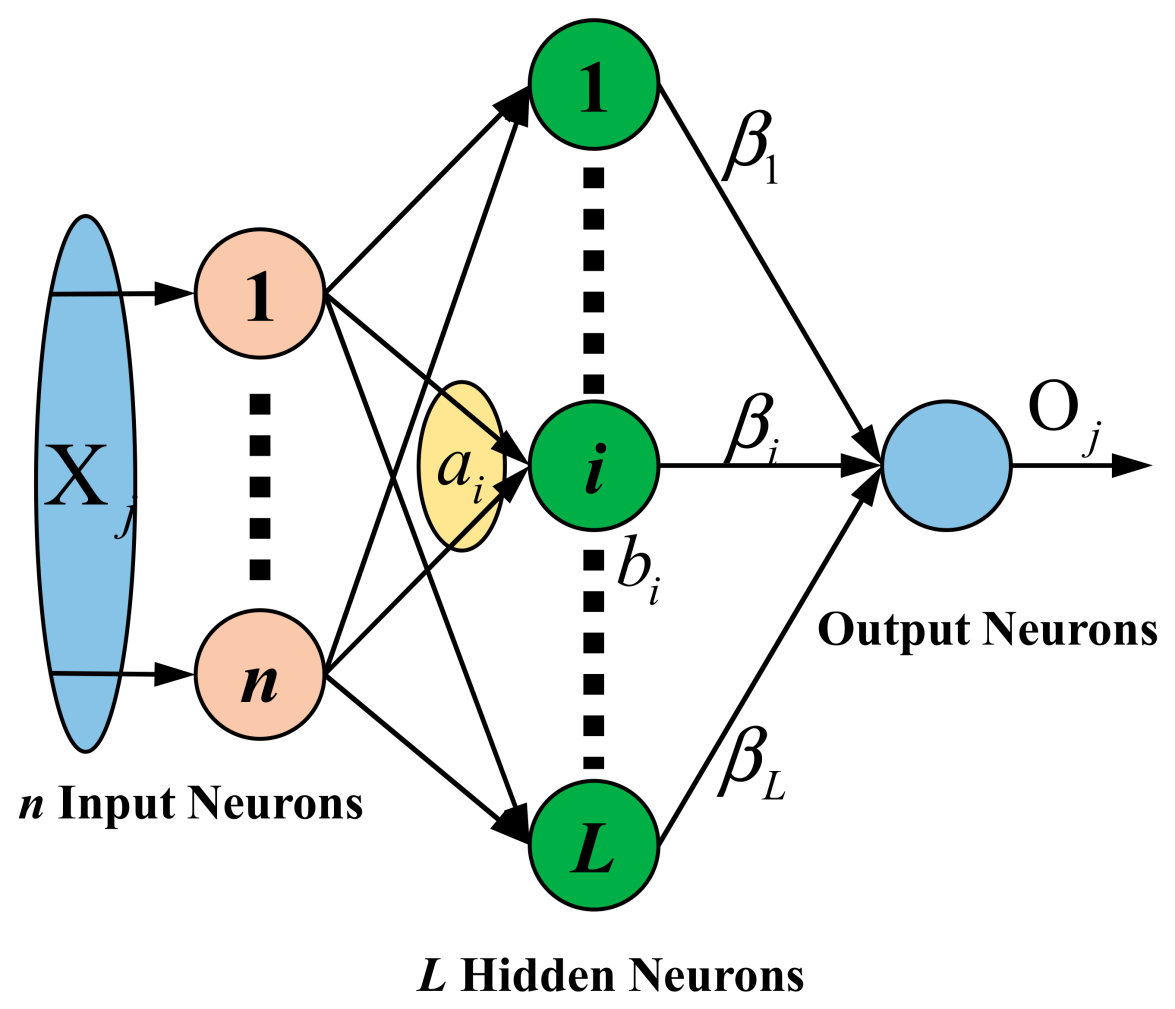
Schematic of signal propagation in the ELM model.

To improve training efficiency relative to traditional back-propagation networks, the extreme learning machine (ELM) was evaluated [29]. ELM is a single-hidden-layer feedforward neural network in which the hidden-layer parameters are initialized without iterative back propagation and the output weights are calculated from the hidden-layer output matrix. This structure provides rapid training, although prediction performance remains sensitive to the hidden-layer configuration and initial parameters. The network structure used in this study is shown in Figure 5.

➀ Normalize the input data.

➁ Oxide-scale thickness, HCl concentration, pickling temperature, and accelerator concentration were used as the four input variables, and pickling time was used as the output variable. Parameter-sensitivity analysis was used to select 24 hidden neurons for the final ELM configuration. Candidate hidden-layer sizes were evaluated only within the inner training folds of the model-selection procedure, so the held-out outer-fold observations did not influence selection of the 24-neuron architecture.

➂ Initialize the connection weights ω between the input and hidden layers and the hidden-neuron biases b. The ReLU function was used as the hidden-layer activation function in the final model configuration. The activation function was selected by the same inner-fold procedure.

➃ Calculate the hidden-layer output matrix H using generalized-inverse matrix theory.

➄ Calculate the connection weights *β* between the hidden layer and the output layers.

➅ Output the predicted pickling time.

**Figure 6:**
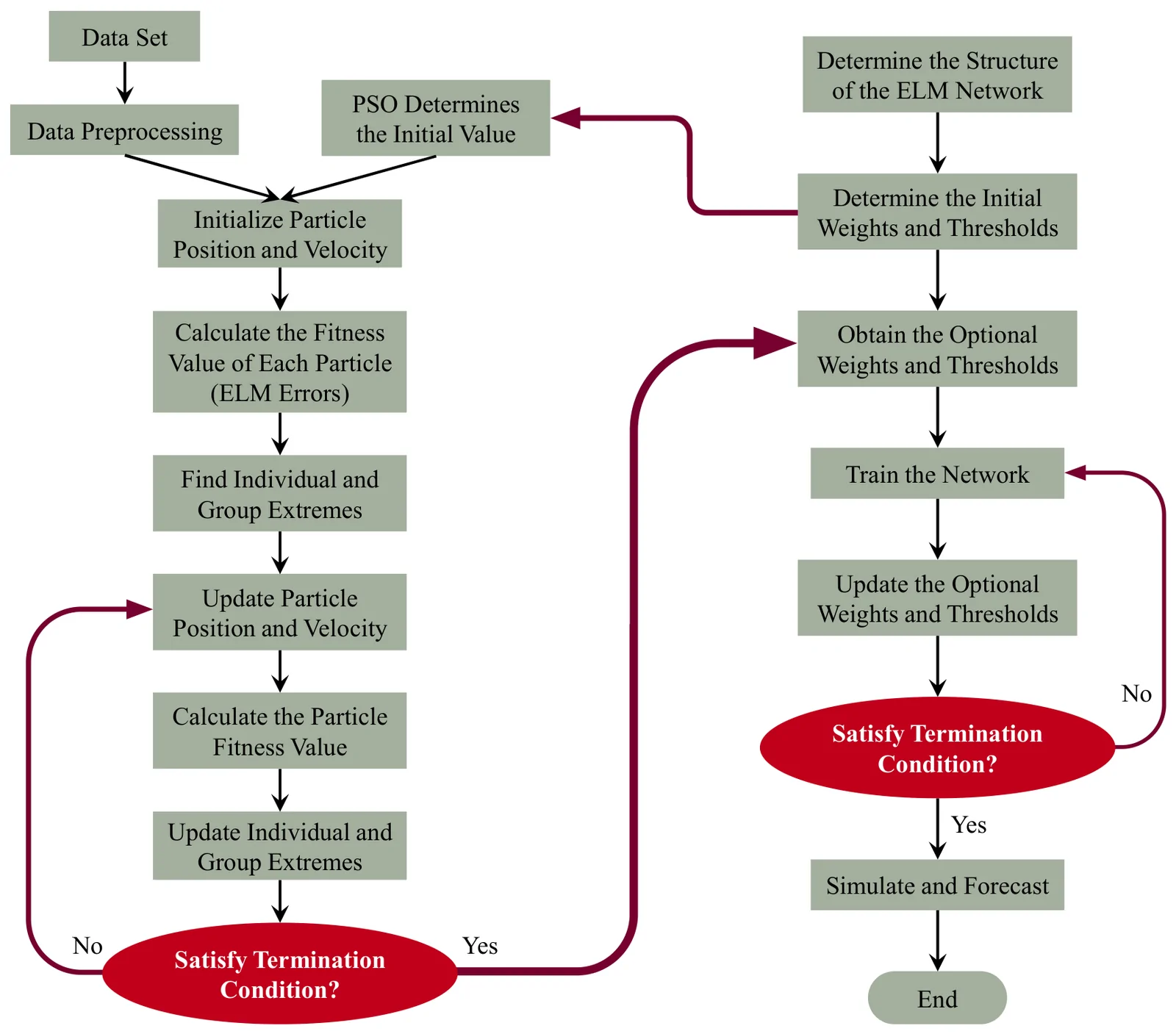
Flow chart of the PSO-ELM modeling procedure.

To reduce the sensitivity of the traditional ELM model to its initial weights and biases, particle swarm optimization (PSO) was used to optimize these parameters [24,25]. PSO updates particle position and velocity according to individual and global best solutions and uses the model error as the fitness criterion. The PSO-ELM modeling procedure is shown in Figure 6.

#### 2.3.3. Statistical Assessment of Machine Learning Models

The modeling dataset contained 48 input–target observations generated from 12 hot-rolled strip groups, each contributing four location-specific records (working-side head, working-side tail, drive-side head, and drive-side tail). Each record contained four inputs—maximum oxide-scale thickness, HCl concentration, pickling temperature, and accelerator concentration—and one target, the pickling time determined by the electrochemical endpoint criterion in Section 2.2. Because four records originating from the same strip group are not statistically independent, the strip group was treated as the grouping unit throughout validation. In the primary six-fold outer allocation, Fold 1 contained strip groups 1 and 7, Fold 2 groups 2 and 8, Fold 3 groups 3 and 9, Fold 4 groups 4 and 10, Fold 5 groups 5 and 11, and Fold 6 groups 6 and 12. Thus, each outer fold contained 8 held-out observations and the remaining 40 observations were used for model development. The complete 48-row input–target dataset and the primary fold assignment are reported directly in Table 2.

To prevent optimistic bias during model selection, all preprocessing and hyperparameter tuning were performed inside the training data of each outer split. Min–max normalization was fitted only to the outer-training data and then applied unchanged to the held-out fold. A five-fold grouped inner cross-validation of the 10 training strip groups was used to select hyperparameters. The grouped six-fold outer assessment was repeated ten times using group-wise shuffling seeds 2026–2035. At every repetition, all four records from one strip group remained together. Reported model metrics are the mean ± standard deviation across the outer-fold/repetition results. This nested grouped procedure ensures that neither scaling parameters nor hyperparameter choices were informed by the samples used for the final performance assessment.

The final settings selected from the inner folds were as follows. BP used a 4–24–1 network with ReLU hidden activation, linear output, learning rate 0.01, and a maximum of 500 epochs. GA-BP used the same network structure with a population size of 80, generation gap 0.80, crossover probability 0.80, mutation probability 0.35, and 500 generations. ELM used 24 hidden neurons with ReLU activation and Moore–Penrose generalized-inverse estimation of output weights. PSO-ELM used 100 particles, Vmax = 0.8, linearly decreasing inertia weight from 0.8 to 0.3, c1 = c2 = 2.8, and 500 iterations. Prediction performance was evaluated using RMSE, MAE, MAPE, and R^2^ [30,31,32]. RMSE and MAE are reported in seconds, and R^2^ is treated as a coefficient of determination rather than as the percentage prediction accuracy.

(1) $$\mathrm{RMSE}=\sqrt{\sum_{i=1}^{n}{\left(Y_{i}-{\hat{Y}}_{i}\right)}^{2}/n}$$

(2) $$\mathrm{MSE}=\sum_{i=1}^{n}\left|Y_{i}-{\hat{Y}}_{i}\right|/n$$

(3) $$\mathrm{MAPE}=\sum_{i=1}^{n}\frac{\left|Y_{i}-{\hat{Y}}_{i}\right|}{Y_{i}}/n$$

(4) $$R^{2}={\left[\frac{\sum_{i=1}^{n}\left({\hat{Y}}_{i}-\bar{\hat{Y}}\right)\left(Y_{i}-\bar{Y}\right)}{\sqrt{\sum_{i=1}^{n}{\left({\hat{Y}}_{i}-\bar{\hat{Y}}\right)}^{2}}\sqrt{\sum_{i=1}^{n}{\left(Y_{i}-\bar{Y}\right)}^{2}}}\right]}^{2}$$

## 3. Results and Discussion

### 3.1. Measurement of Oxide-Scale Thickness

**Figure 7:**
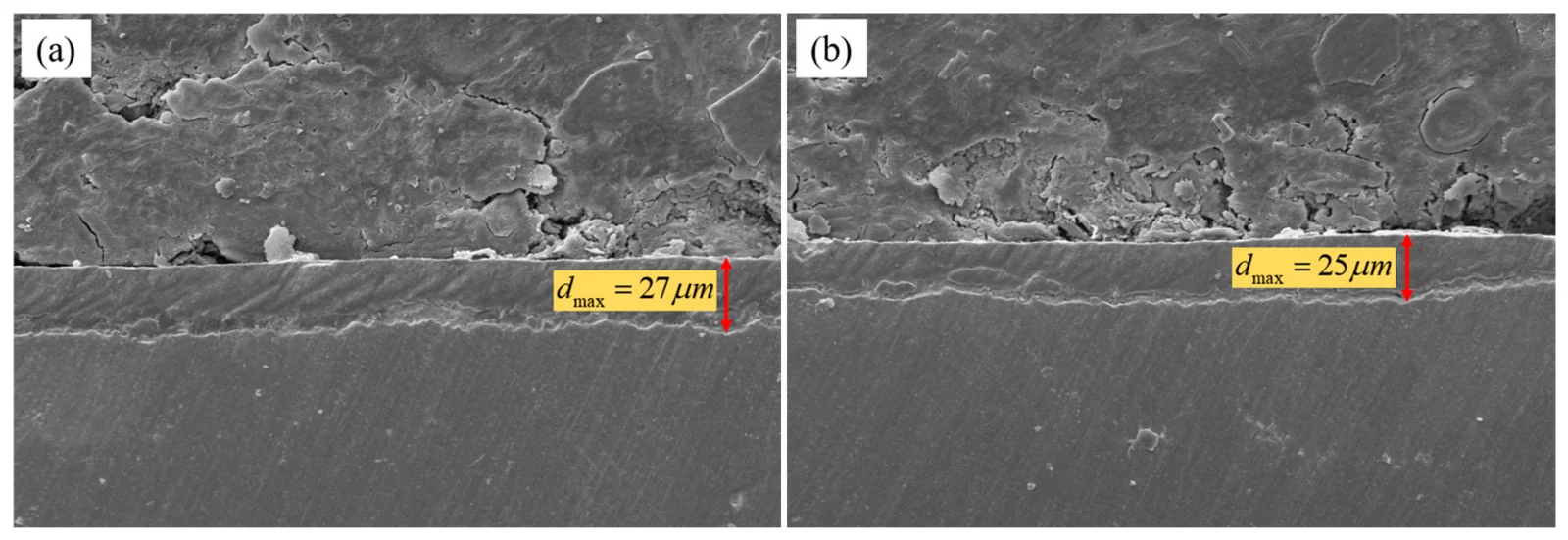
Representative cross-sectional SEM images of the oxide scale: (**a**) strip edge; and (**b**) strip middle.

Before pickling, oxide-scale thickness was measured to establish its relationship with pickling time. Scanning electron microscopy (SEM) was used for quantitative cross-sectional characterization because it provided higher spatial resolution than the preliminary optical observations described in Section 2.1. Because complete scale removal is controlled by the locally thickest region, the maximum observed thickness was used to characterize each sampled location. Representative SEM images from the edge and middle regions of the hot-rolled strip are shown in Figure 7.

Figure 7 shows that the oxide scale at the strip edge was comparatively continuous, dense, and uniform, whereas the middle region showed greater thickness non-uniformity. The edge region was therefore selected as the principal location for the pickling study. The strip edges were classified as working side and drive side, and thickness specimens were obtained from the head and tail positions. Table 3 summarizes the oxide-scale thickness ranges measured at the four locations for 12 hot-rolled strip groups.

Table 3 shows appreciable variation in oxide-scale thickness among the four sampling locations. The location-specific observations from a given strip group share the same production history and are therefore not treated as independent samples during validation. All four records from each strip group were assigned to the same outer fold, as listed in Table 2.

### 3.2. Effect of Pickling Parameters on Pickling Time

**Figure 8:**
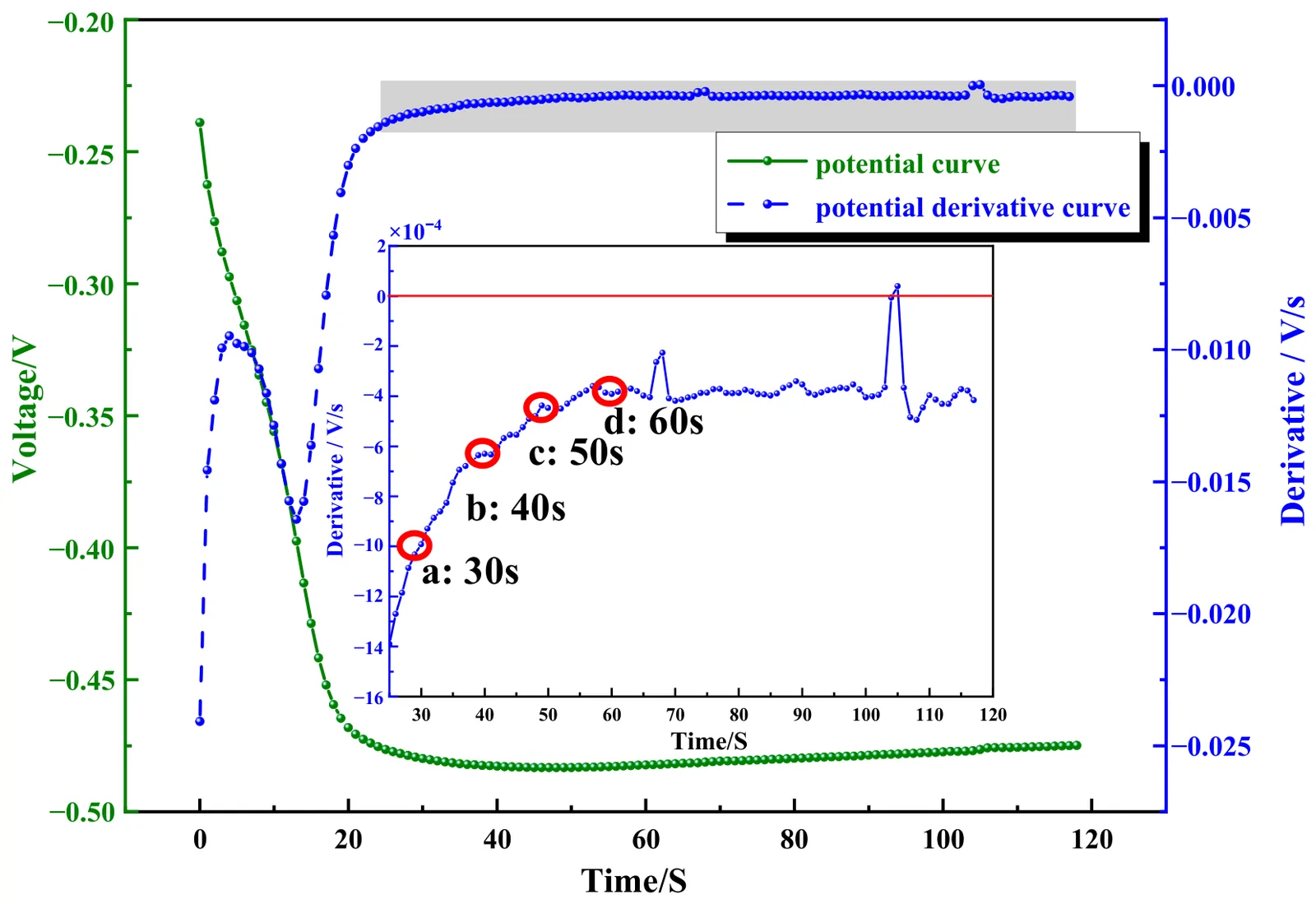
Open-circuit-potential curve and potential-derivative curve for a representative pickling condition; potential derivative is reported in V/s.

**Figure 9:**
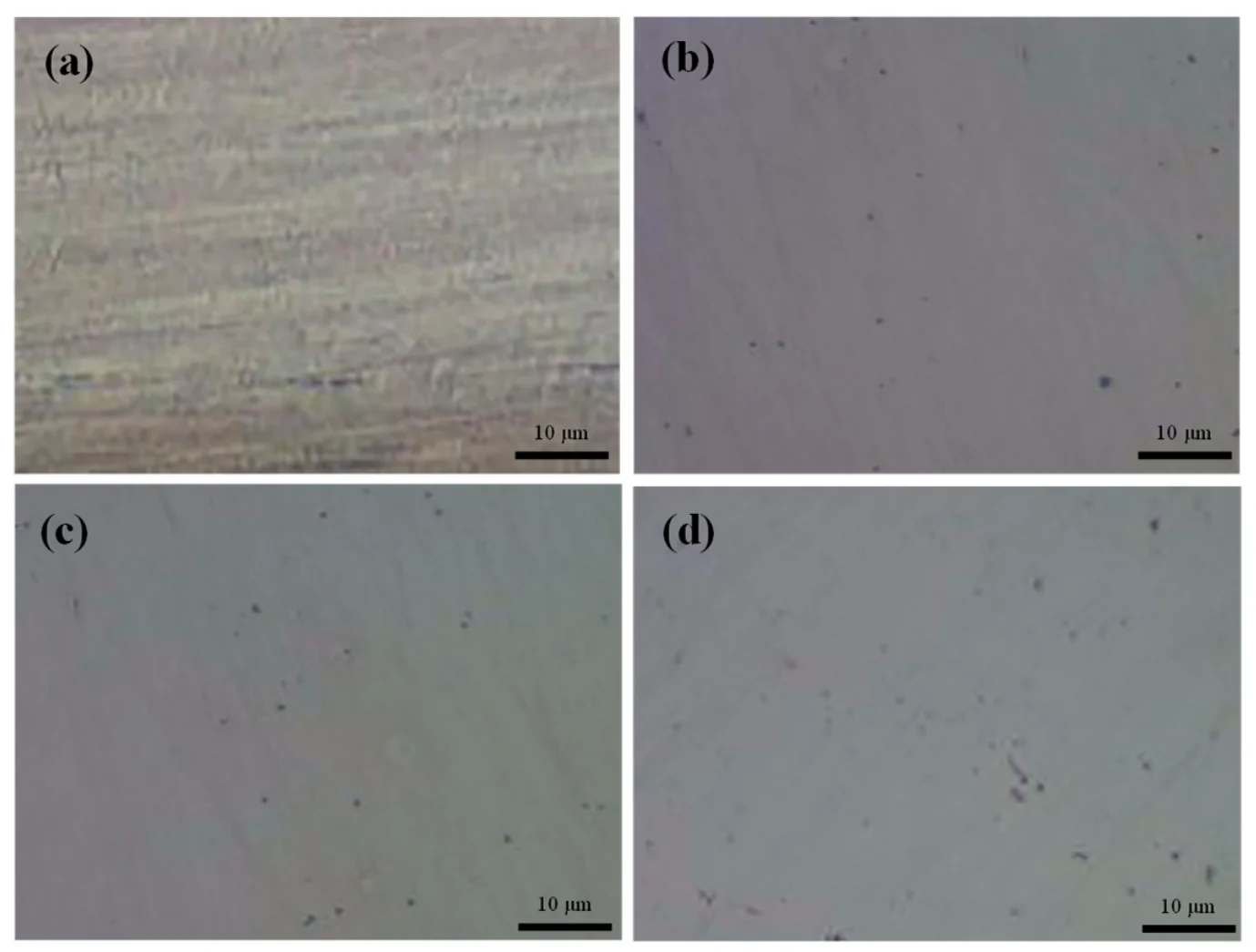
Surface condition of the hot-rolled strip after different pickling times: (**a**) 30 s; (**b**) 40 s; (**c**) 50 s; and (**d**) 60 s.

The key step in the potential-derivative near-zero method is identification of the operational endpoint. Figure 8 shows the potential curve and its derivative for a representative condition of 6% HCl, 75 °C, and no accelerator. Starting from 20 s, specimens were removed at 10 s intervals, rinsed, dried, and examined by optical microscopy. The surface states after 30, 40, 50, and 60 s are shown in Figure 9.

**Figure 10:**
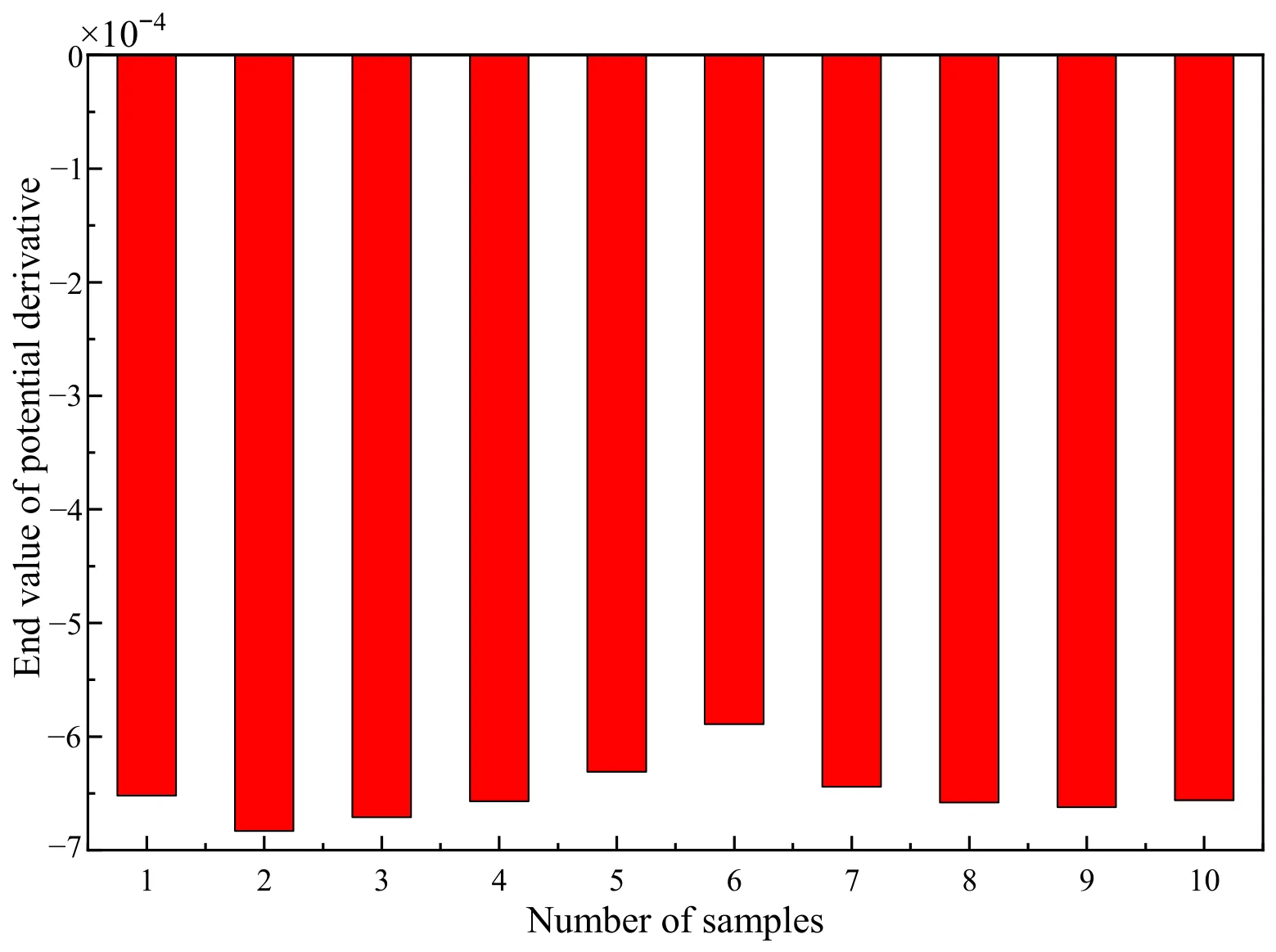
Mean potential-derivative values at microscopy-defined completion under ten representative pickling conditions (n = 3). Bars show the mean values, and the corresponding ±1 SD values are reported in Table 4.

Under the representative condition shown in Figure 8 and Figure 9, the potential derivative first reached zero only at approximately 105 s. Waiting for this exact-zero point would therefore extend exposure well beyond the time required for oxide-scale removal. By contrast, the surface showed little further change after approximately 40 s, and the derivative at 40 s was −6.52 × 10^−4^ V/s. To make the surface criterion objective, the Figure 9 micrographs were background-normalized and segmented using a single grayscale threshold after removal of labels and scale bars. The residual-scale area fraction decreased from 36.8% at 30 s to 2.3%, 1.4%, and 1.1% at 40, 50, and 60 s, respectively. Using a residual-scale area fraction of 3% as the microscopy completion criterion therefore identifies approximately 40 s as the completion time under this representative condition. This quantitative image analysis confirms that oxide-scale removal precedes the time at which $\frac{dE}{dt}$ becomes exactly zero. The critical values of the potential-derivative curves under different pickling processes were measured in the same way and summarized in Figure 10. Each bar in Figure 10 represents the mean potential-derivative value obtained from three independent tests under the corresponding condition, and the associated ±1 SD values are reported in Table 4.

Across the ten conditions, microscopy-observed completion occurred while the potential derivative remained more negative than the adopted near-zero threshold. The least-negative mean derivative at observed completion was −5.89 ± 0.14 × 10^−4^ V/s. Even the upper bound estimated as mean + 2SD (−5.61 × 10^−4^ V/s) remained more negative than −5 × 10^−4^ V/s. Accordingly, −5 × 10^−4^ V/s provides a conservative common endpoint: it is reached after microscopy indicates scale removal, thereby reducing the probability of under-pickling while avoiding the much longer exposure required for $\frac{dE}{dt}$ = 0. The pickling time used in the subsequent process analysis and machine-learning dataset was defined by this operational threshold.

**Figure 11:**
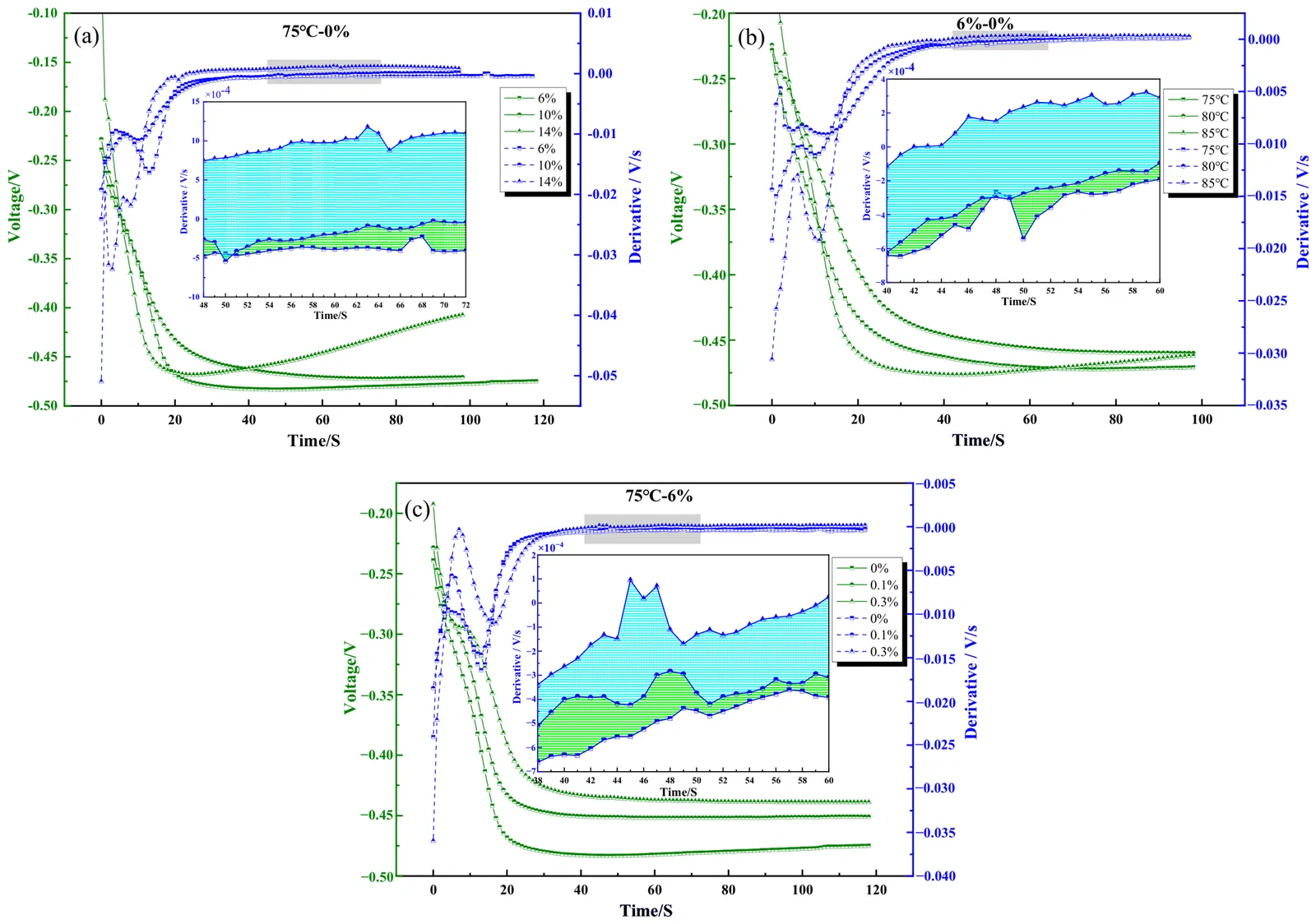
Open-circuit-potential and potential-derivative curves under different pickling conditions: (**a**) effect of HCl concentration; (**b**) effect of pickling temperature; and (**c**) effect of accelerator concentration.

**Figure 12:**
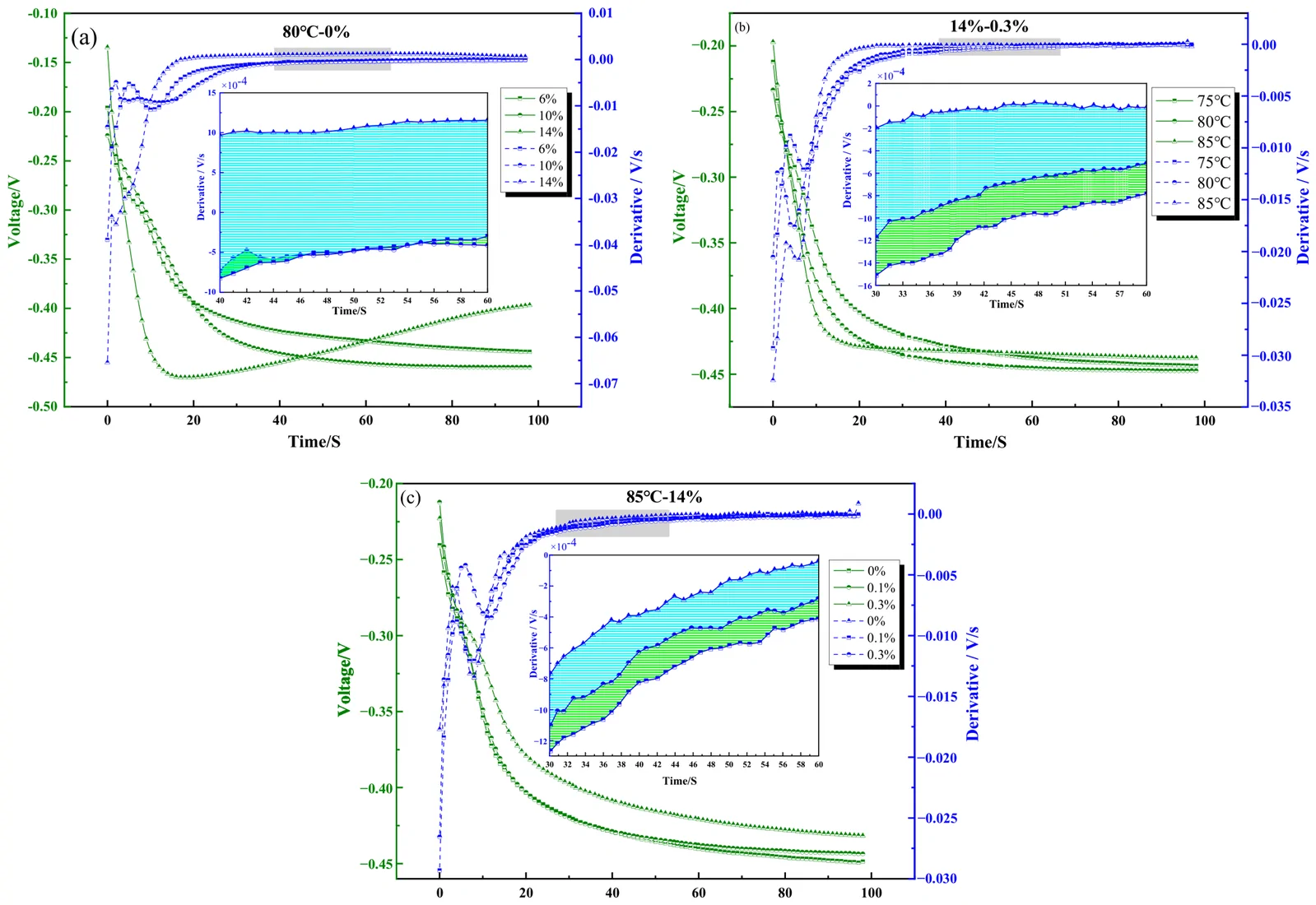
Open-circuit-potential and potential-derivative curves after removal of detached oxide scale: (**a**) effect of HCl concentration; (**b**) effect of pickling temperature; and (**c**) effect of accelerator concentration.

Accordingly, the operational endpoint for subsequent experiments was defined as the time at which the potential derivative first reached −5 × 10^−4^ V/s and satisfied the five-consecutive-sample persistence criterion described in Section 2.2. This threshold was selected because it was reached after microscopy indicated completion of oxide-scale removal, thereby reducing the risk of under-pickling while avoiding the substantially longer exposure required for $\frac{dE}{dt}$ = 0. Because substrate dissolution was not quantitatively evaluated in this study, this criterion should not be interpreted as demonstrating the complete absence of over-pickling. Based on this endpoint criterion, the effects of HCl concentration, pickling temperature, and accelerator concentration on pickling time were examined using a controlled-variable design with a representative maximum oxide-scale thickness of 27 ± 1 μm. For each process condition, three independent pickling experiments were performed. To avoid overplotting, the curve shown for each condition in Figure 11 and Figure 12 corresponds to the replicate whose electrochemically determined endpoint time was closest to the mean endpoint time of the three replicates.

Figure 11 shows that, within the tested ranges, pickling time generally decreased as HCl concentration, temperature, or accelerator concentration increased. Under the illustrated condition, increasing HCl concentration from 6% to 10% produced a smaller change in pickling time than increasing it from 10% to 14%. Similarly, the reduction in pickling time from 80 to 85 °C was more pronounced than that from 75 to 80 °C. The accelerator also shortened the pickling time over the tested range. These qualitative trends are consistent with the dependence of iron-oxide dissolution on acid concentration and temperature reported for hydrochloric-acid dissolution of scale [17]. The present experiments, however, were limited to 6–14% HCl and 75–85 °C and therefore do not establish universal requirements such as a HCl concentration below 15% or temperature below 90 °C. Production cost, worker exposure, and final product-quality improvements were not measured directly and are therefore not treated as experimentally demonstrated outcomes. Additional tests under other combinations of the investigated parameters produced the same qualitative trends. During these tests, fluctuations were observed in the potential-derivative curves. Because oxide-scale detachment during pickling can disturb the electrochemical signal, the detached scale was removed during a separate set of measurements and the resulting curves are shown in Figure 12. Hemmelmann et al. [17] similarly reported that iron-oxide dissolution in hydrochloric acid is strongly dependent on acid concentration and temperature, supporting the direction of the present trends. Colla et al. [18] also emphasized the sensitivity of under-pickling defects to process conditions. The accelerator effect observed here is discussed qualitatively rather than compared numerically with the literature values because commercial accelerator chemistry and dosage bases vary substantially among pickling systems.

Comparison of Figure 11 and Figure 12 shows that removing detached oxide scale produced smoother potential-derivative curves while preserving the qualitative effects of HCl concentration, temperature, and accelerator concentration. This supports the interpretation that loose-scale detachment contributes to fluctuations in the electrochemical signal. Automatic management of detached scales in an industrial pickling line remains a process-engineering issue beyond the scope of the present laboratory experiments.

### 3.3. Comparison of Various Hybrid Machine Learning Approaches

**Figure 13:**
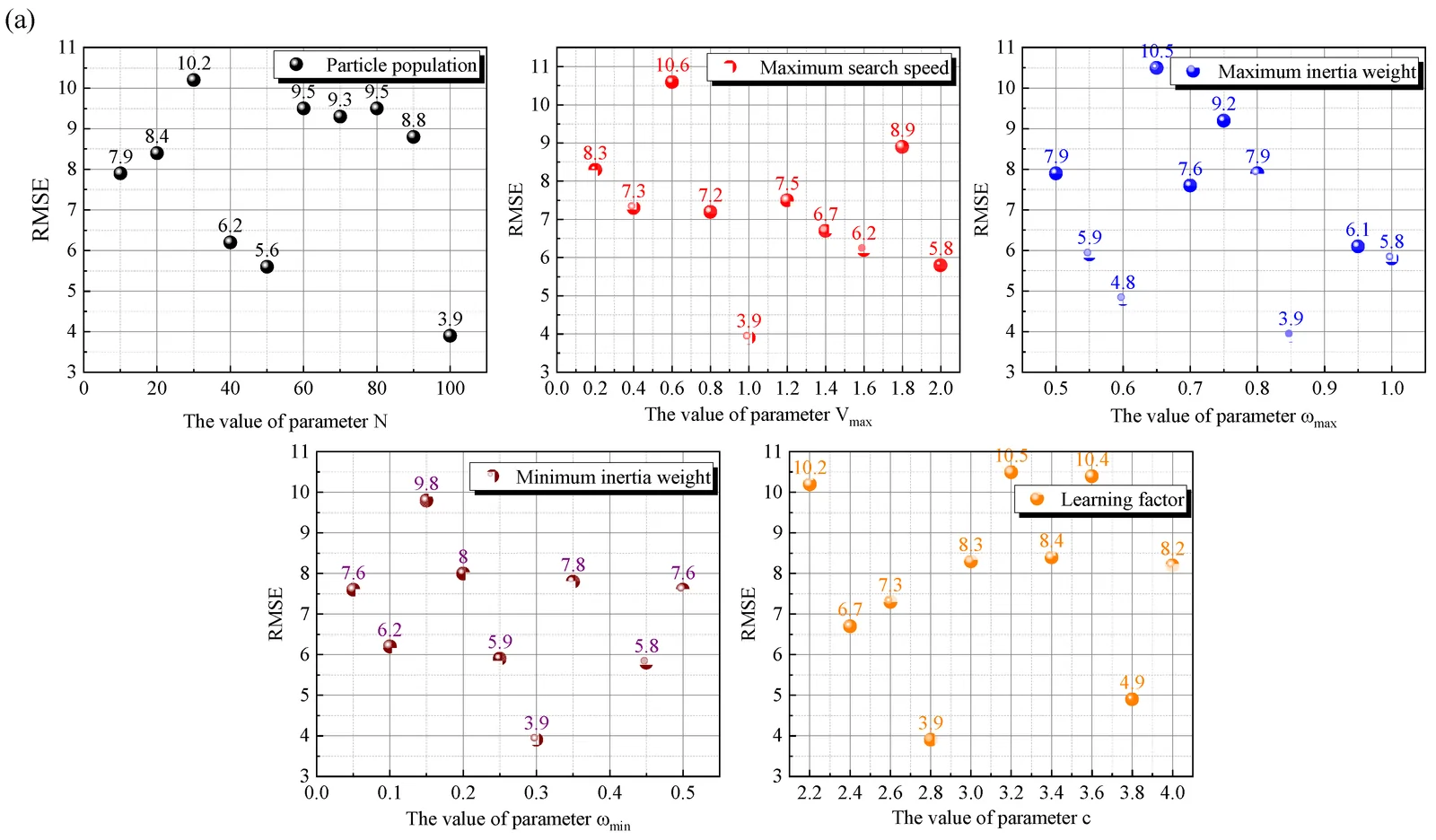

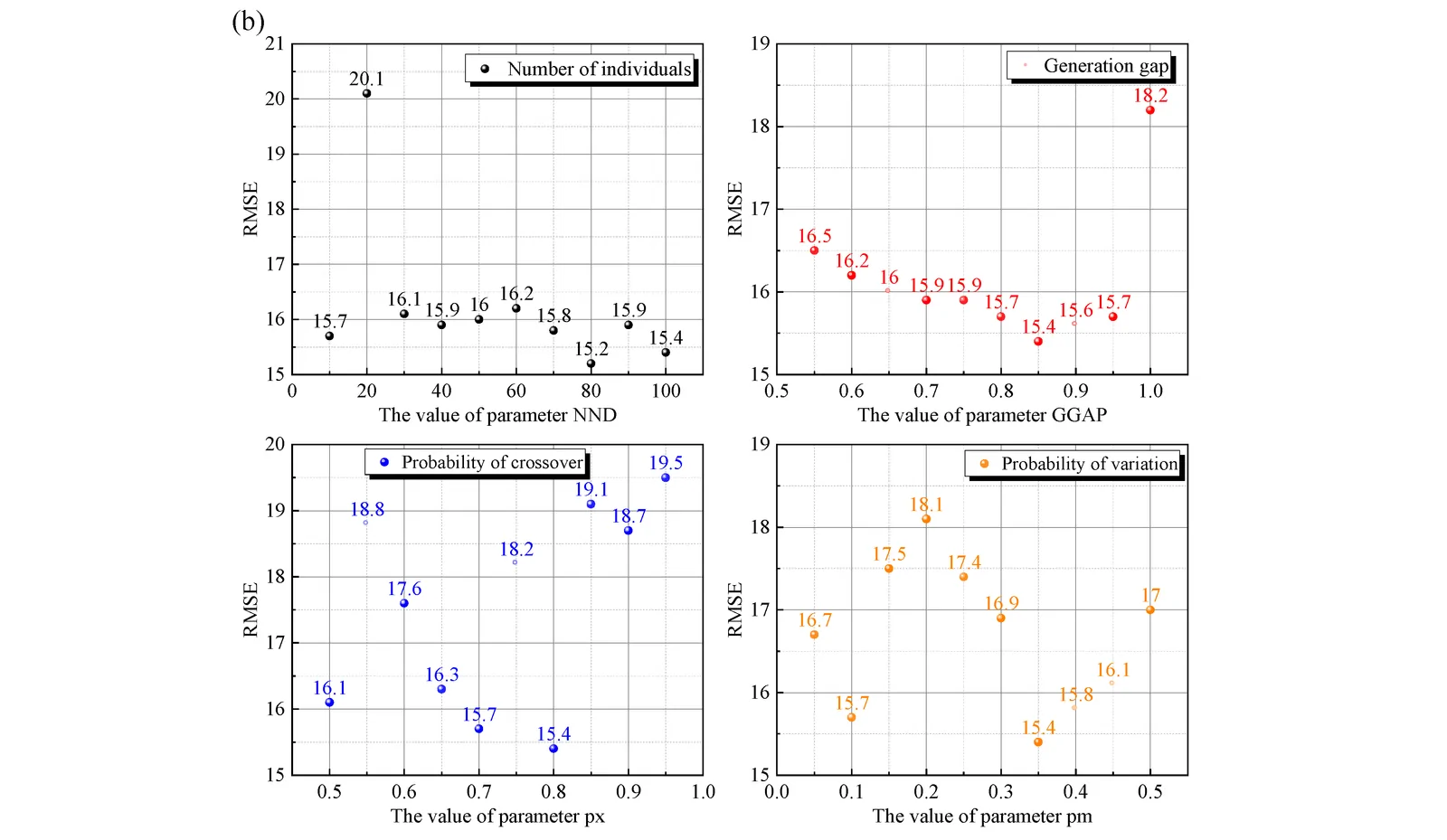
Effect of model-parameter variation on training RMSE: (**a**) PSO-ELM model; and (**b**) GA-BP model.

The relationship between oxide-scale thickness, pickling parameters, and pickling time is nonlinear, making simple linear regression insufficient for the present dataset. Four machine-learning regression models—BP, GA-BP, ELM, and PSO-ELM—were therefore evaluated. GA and PSO were used to reduce the sensitivity of BP and ELM, respectively, to their initial parameters. Parameter sensitivity was examined for the optimized models while the corresponding unoptimized model settings were kept comparable. RMSE was used to assess the effect of model-parameter changes, as summarized in Figure 13.

**Figure 14:**
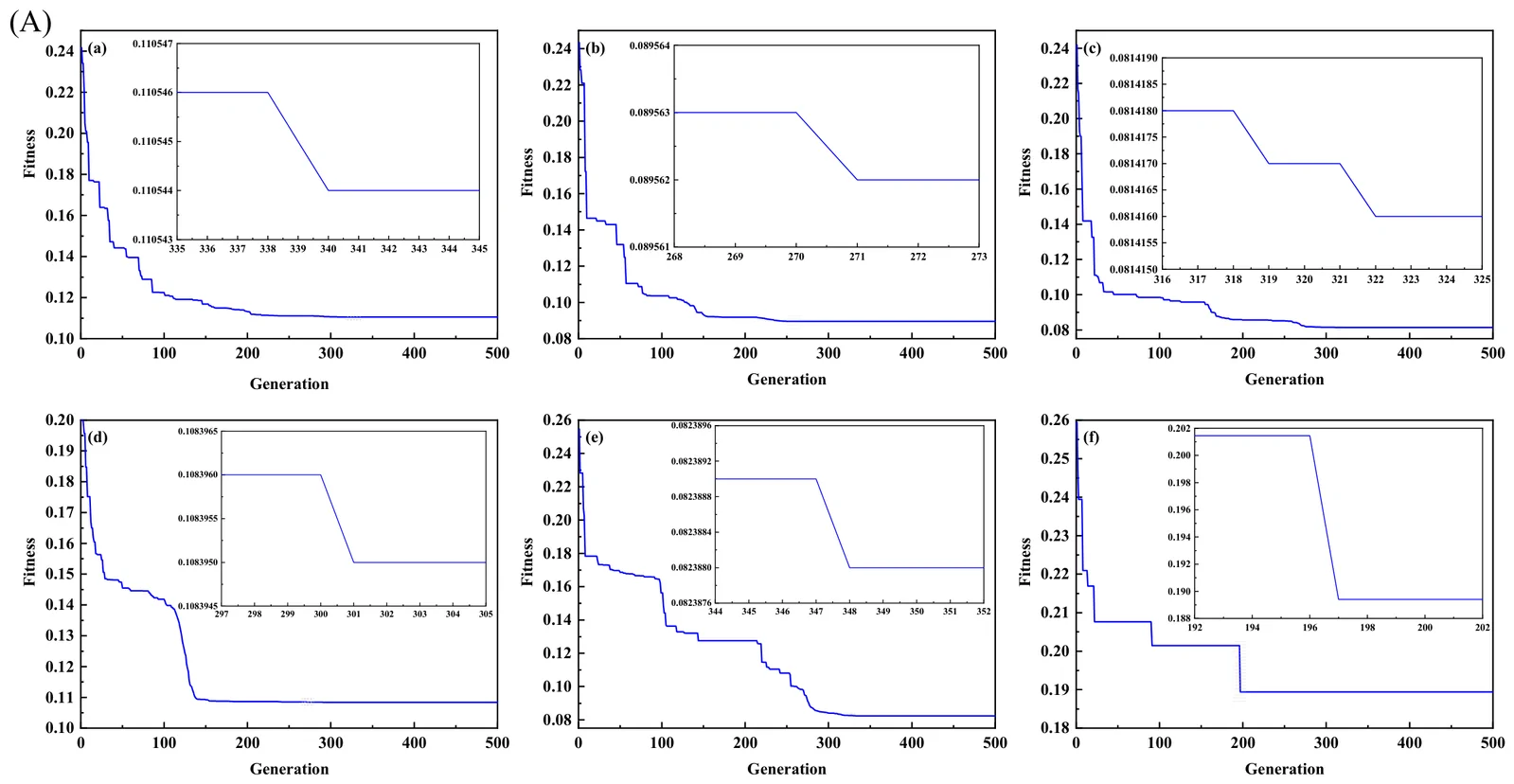

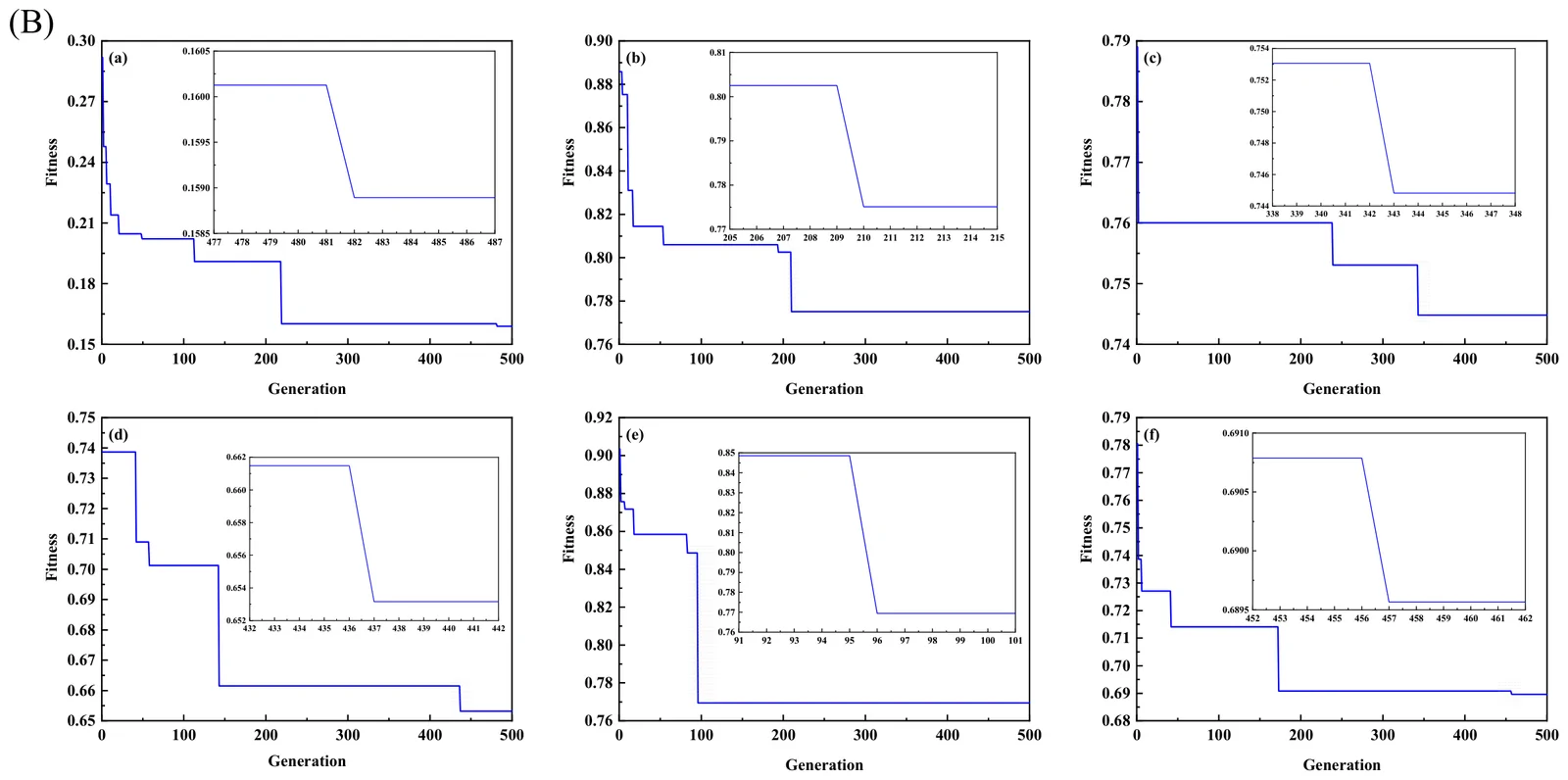
Fitness evolution curve: (**A**) PSO-ELM model: (**a**) 1-fold; (**b**) 2-fold; (**c**) 3-fold; (**d**) 4-fold; (**e**) 5-fold; (**f**) 6-fold; and (**B**) GA-BP model l: (**a**) 1-fold; (**b**) 2-fold; (**c**) 3-fold; (**d**) 4-fold; (**e**) 5-fold; (**f**) 6-fold.

Figure 13 shows that model-parameter selection strongly affected the training error. The minimum training RMSE obtained for PSO-ELM was lower than that obtained for GA-BP, indicating a more favorable optimized configuration for the present dataset. After the model parameters were fixed, the final comparisons were performed using the six-fold cross-validation procedure described in Section 2.3.3. Mean squared error (MSE) was used as the fitness function during optimization, and the maximum number of evolutionary iterations was set to 500. The fitness-evolution curves for PSO-ELM and GA-BP are shown in Figure 14. Across the folds, PSO-ELM generally converged to lower fitness values than GA-BP. In the revised validation workflow, the parameter ranges illustrated in Figure 13 were searched within the grouped inner folds only, and the outer held-out fold was not used to choose these settings.

**Figure 15:**
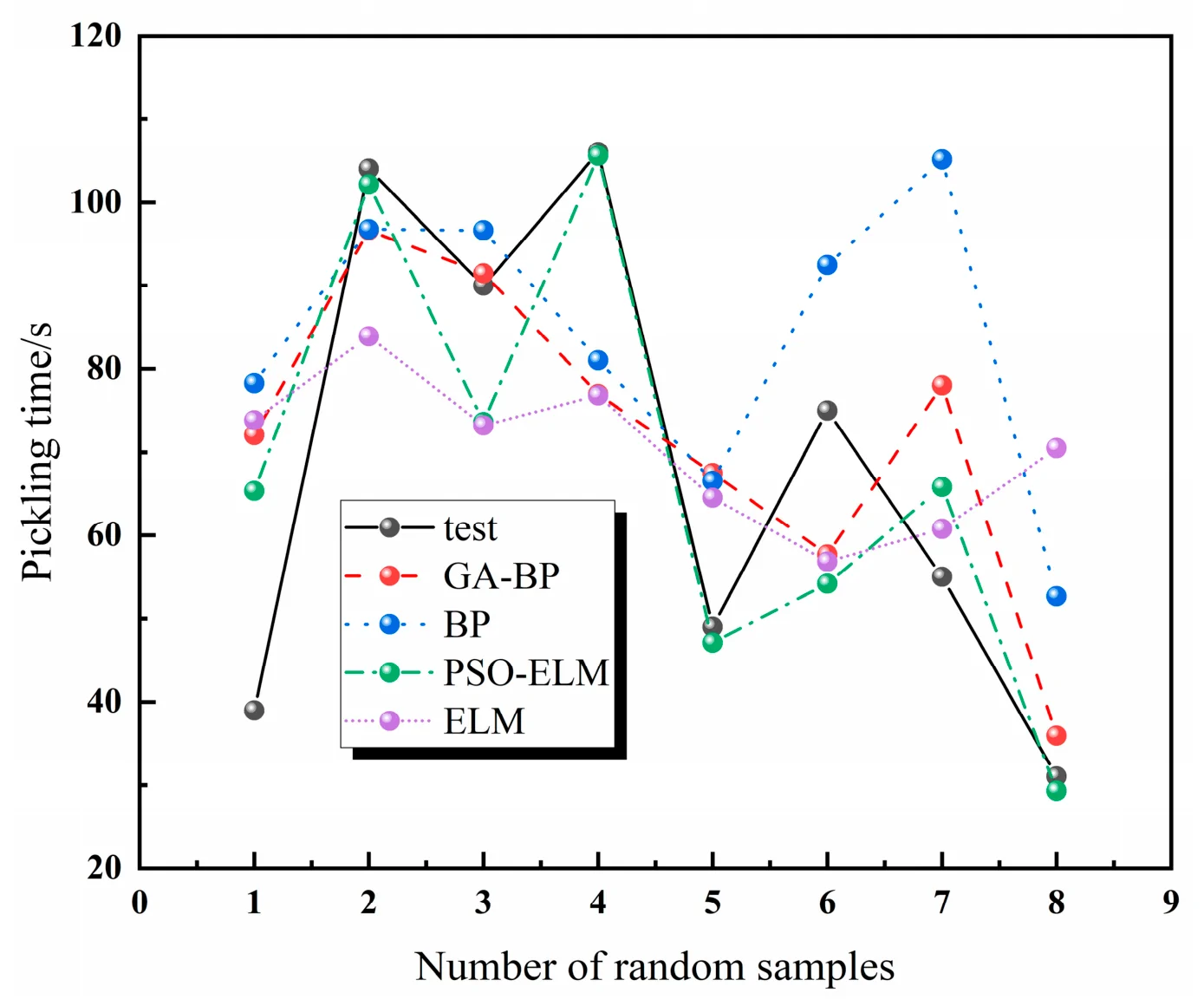
Measured and predicted pickling times for one representative eight-sample cross-validation test fold using BP, GA-BP, ELM, and PSO-ELM models.

To illustrate prediction behavior, Figure 15 presents the measured pickling times and predictions from BP, GA-BP, ELM, and PSO-ELM for the eight held-out observations in the representative primary Fold 1 listed in Table 2. The black curve denotes the measured values and the other curves denote model predictions. This figure is provided only as a visual example of one outer test fold, and model selection and quantitative conclusions are based on the repeated grouped nested cross-validation results summarized in Table 5.

The eight observations displayed in Figure 15 are the held-out observations from the representative Fold 1 and are not an independent external validation dataset Figure 15 shows that PSO-ELM follows the measured values closely for several samples, although noticeable deviations remain for some cases. GA-BP also follows the overall trend but produces larger deviations for some observations. Because visual agreement in a single eight-sample fold is insufficient for model selection, all four models were evaluated from the complete repeated grouped outer-fold predictions using R^2^, MAE, MAPE, and RMSE. The resulting distributions are summarized as mean ± SD in Table 5.

Table 5 shows that PSO-ELM achieved the best prediction performance among the four evaluated models, with R^2^ = 0.85 ± 0.05, MAE = 7.27 ± 1.01 s, MAPE = 0.14 ± 0.02, and RMSE = 9.65 ± 1.48 s. Across the outer-fold/repetition results, PSO-ELM R^2^ ranged from approximately 0.78 to 0.90 and RMSE from 7.6 to 11.8 s, indicating that its advantage was not confined to a single favorable split. GA-BP ranked second, whereas the unoptimized BP and ELM models produced larger errors. R^2^ is a coefficient of determination. Therefore, R^2^ = 0.85 is not interpreted as 85% prediction accuracy. The improved performance of optimization-assisted ELM is consistent with the use of PSO-ELM in other nonlinear engineering-prediction problems [28,29], while machine-learning studies in steel processing have likewise demonstrated the value of process variables for predicting manufacturing outcomes [6,7]. Direct numerical comparison with those studies is not appropriate because the predicted quantities, datasets, and validation protocols differ. Relative to our earlier terahertz study [23], the present work predicts a different target—electrochemically defined pickling time—using oxide-scale thickness as one of the inputs. The present results should therefore be interpreted as a proof of concept for the investigated DP600 steel and process ranges. The dataset contains 48 observations from 12 strip groups, oxide-scale thickness is spatially non-uniform, and uncertainty in endpoint labeling propagates into model training. Larger multi-coil and multi-grade datasets, independent production campaigns, and revalidation of the electrochemical endpoint on the target measurement system are required before direct industrial process-control deployment.

## 4. Conclusions

This study combined electrochemical endpoint determination with machine-learning regression to investigate pickling time for hot-rolled automotive DP600 steel. Open-circuit-potential monitoring showed that waiting for the potential derivative to reach exactly zero can substantially extend exposure after oxide-scale removal. A signal-processing protocol consisting of 1 s acquisition, five-point second-order Savitzky–Golay smoothing, central finite-difference differentiation, and a five-sample persistence rule was therefore used with an operational endpoint threshold of −5 × 10^−4^ V/s. Triplicate tests across ten representative process conditions and quantitative microscopy analysis supported the threshold within the investigated window. Within 6–14 wt.% HCl, 75–85 °C, and 0–3‰ accelerator, increasing each process variable generally reduced pickling time. No universal process limit outside these tested ranges is inferred. Four regression models were assessed using repeated leakage-free grouped nested cross-validation. PSO-ELM provided the best mean performance, with R^2^ = 0.85 ± 0.05, MAE = 7.27 ± 1.01 s, MAPE = 0.14 ± 0.02, and RMSE = 9.65 ± 1.48 s. These results show that oxide-scale thickness and controllable pickling variables can support data-driven estimation of pickling time, but the current study remains limited to 48 observations from 12 strip groups and one DP steel grade. Spatial scale non-uniformity and endpoint uncertainty remain important sources of prediction variance. Accordingly, the method should be regarded as a laboratory-scale proof of concept pending validation on larger multi-grade datasets and independent industrial production campaigns.

## Figures and Tables

**Table 1:** Pickling parameters and tested levels.

Factor	Tested Levels
Temperature/°C	75, 80, 85
HCl concentration/wt.%	6, 10, 14
Accelerator concentration/‰	0, 1, 3

**Table 2:** Complete machine-learning input–target dataset and primary grouped outer-fold allocation. Site abbreviations: WH, working-side head; WT, working-side tail; DH, drive-side head; and DT, drive-side tail.

Num	Strip	Site	d_max_/μm	HCl/wt.%	T/°C	Accel./‰	Pickling Time/s	Fold
1	1	WH	26	14	85	3	38.0	1
2	1	WT	28	6	75	0	103.0	1
3	1	DH	28	6	80	0	91.0	1
4	1	DT	26	6	75	0	105.0	1
5	2	WH	26	6	75	0	87.3	2
6	2	WT	24	10	75	0	77.7	2
7	2	DH	28	14	75	0	52.7	2
8	2	DT	27	6	80	0	90.8	2
9	3	WH	25	6	85	0	69.1	3
10	3	WT	29	6	75	1	84.5	3
11	3	DH	24	6	75	3	73.2	3
12	3	DT	28	10	80	1	62.6	3
13	4	WH	26	14	80	1	47.7	4
14	4	WT	22	14	85	3	32.3	4
15	4	DH	25	10	85	0	60.5	4
16	4	DT	24	14	75	1	62.9	4
17	5	WH	26	6	75	0	88.6	5
18	5	WT	24	10	75	0	74.2	5
19	5	DH	26	14	75	0	58.6	5
20	5	DT	23	6	80	0	77.2	5
21	6	WH	24	6	85	0	68.8	6
22	6	WT	23	6	75	1	89.4	6
23	6	DH	27	6	75	3	81.0	6
24	6	DT	25	10	80	1	58.1	6
25	7	WH	25	14	80	1	50.0	1
26	7	WT	24	10	75	0	74.0	1
27	7	DH	27	10	80	1	52.0	1
28	7	DT	23	14	85	3	28.0	1
29	8	WH	29	14	80	1	44.7	2
30	8	WT	26	14	85	3	34.9	2
31	8	DH	27	10	85	0	53.5	2
32	8	DT	24	14	75	1	55.9	2
33	9	WH	25	6	75	0	95.1	3
34	9	WT	28	10	75	0	89.4	3
35	9	DH	26	14	75	0	58.7	3
36	9	DT	28	6	80	0	93.3	3
37	10	WH	26	6	85	0	62.4	4
38	10	WT	29	6	75	1	87.6	4
39	10	DH	28	6	75	3	72.9	4
40	10	DT	28	10	80	1	68.3	4
41	11	WH	24	14	80	1	52.4	5
42	11	WT	24	14	85	3	40.7	5
43	11	DH	25	10	85	0	54.3	5
44	11	DT	28	14	75	1	67.3	5
45	12	WH	26	6	75	0	88.6	6
46	12	WT	28	10	75	0	77.1	6
47	12	DH	26	14	75	0	62.2	6
48	12	DT	30	6	80	0	90.4	6

**Table 3:** Oxide-scale thickness ranges at different locations of the hot-rolled strip.

Num	Working-Side Head/μm	Working-Side Tail/μm	Drive-Side Head/μm	Drive-Side Tail/μm
1	22–26	23–28	24–28	23–26
2	23–26	22–24	25–28	25–27
3	22–25	28–29	20–24	26–28
4	23–26	19–22	22–25	20–24
5	25–26	21–24	25–26	21–23
6	22–24	21–23	24–27	23–25
7	22–25	20–24	25–27	19–23
8	28–29	23–26	24–27	22–24
9	22–25	26–28	23–26	23–28
10	23–26	28–29	25–28	24–28
11	20–24	22–24	22–25	24–28
12	25–26	26–28	23–26	27–30

**Table 4:** Validation of the potential-derivative endpoint under representative pickling conditions (n = 3, mean ± SD).

Num	HCl/wt.%	Temperature/°C	Accelerator/‰	Microscopy Completion Time/s	$\frac{dE}{dt}$ at Completion/×10^−4^ V/s
1	6	75	0	40.0 ± 1.0	−6.52 ± 0.18
2	10	75	0	38.0 ± 1.0	−6.84 ± 0.21
3	14	75	0	34.3 ± 1.5	−6.70 ± 0.16
4	6	80	0	38.3 ± 1.2	−6.57 ± 0.17
5	6	85	0	34.7 ± 1.2	−6.32 ± 0.15
6	6	75	1	36.7 ± 1.5	−5.89 ± 0.14
7	6	75	3	32.3 ± 1.2	−6.44 ± 0.19
8	10	80	1	31.0 ± 1.0	−6.59 ± 0.16
9	14	80	1	27.7 ± 1.2	−6.62 ± 0.18
10	14	85	3	23.7 ± 1.2	−6.57 ± 0.17

**Table 5:** Repeated grouped nested cross-validation performance of BP, GA-BP, ELM, and PSO-ELM models (mean ± SD across outer folds/repetitions; MAE and RMSE in s).

Prediction Performance	R^2^	MAE	MAPE	RMSE
BP model	0.34 ± 0.12	18.81 ± 3.20	0.37 ± 0.08	24.62 ± 4.10
GA-BP model	0.65 ± 0.09	12.45 ± 2.30	0.23 ± 0.05	15.68 ± 2.75
ELM model	0.19 ± 0.15	22.95 ± 4.10	0.49 ± 0.10	25.94 ± 5.10
PSO-ELM model	0.85 ± 0.05	7.27 ± 1.01	0.14 ± 0.02	9.65 ± 1.48

## Data Availability

The original contributions presented in this study are included in the article. Further inquiries can be directed to the corresponding authors.
